# Retained Primary Teeth and Their Complication and Treatment: A Case Report

**DOI:** 10.7759/cureus.67197

**Published:** 2024-08-19

**Authors:** Ruchi S Alizar (Jain), Neha Pankey, Priyanka Paul

**Affiliations:** 1 Department of Pedodontics and Preventive Dentistry, Sharad Pawar Dental College and Hospital, Datta Meghe Institute of Higher Education and Research, Wardha, IND; 2 Department of Public Health Dentistry, Sharad Pawar Dental College and Hospital, Datta Meghe Institute of Higher Education and Research, Wardha, IND

**Keywords:** over-retained primary teeth, peri-radicular cyst, extraction, dental caries, dental trauma, exfoliation time

## Abstract

In dentistry, over-retained deciduous teeth pose a distinct issue due to their endurance during the expected exfoliation period. This case study describes a clinical situation with a male patient who is seven years old and complaining of painful swelling over the maxillary anterior region of the jaw and clinically showing dark red-colored, firm, non-tender, purulent swelling extending from alveolar mucosa of the upper anterior to the gingival margin of the 51, 52, 53 region. The treatment plan includes the extraction of the primary teeth. This case report also highlights the importance of minimal surgical intervention for treating peri-radicular cysts to minimize healing time and damage to the underlying developing permanent tooth.

## Introduction

The primary teeth are important for aesthetics, mastication, phonetics, and normal development of occlusion in permanent dentition [1]. The primary teeth may remain for a a number of causes, the most typical being the developmental absence of the permanent successor [2]. Over-retention of deciduous teeth is a dental anomaly in which one or more primary maxillary anterior teeth persist beyond their expected exfoliation time, i.e., six years as in our case. This disorder can change the eruption route of permanent teeth and prevent them from erupting properly [3]. Several dental issues can arise from this condition, including delayed eruption of permanent teeth, caries, crowding, malocclusions, and ankylosis [4].

Exfoliation of disturbed primary or deciduous teeth may occur early or late. The precise process of primary tooth exfoliation may involve the periodontal ligament's monocytes differentiating into odontoclasts or pressure resorption of the deciduous root caused by the successional tooth that is erupting. The deciduous root is then reabsorbed by the odontoclasts without causing an inflammatory reaction, much like osteoclasts do during bone remodeling or resorption [5]. The factor or factors that initiate this process are still unknown.

In this case scenario, there is over-retention of the primary anterior teeth due to trauma, which was left untreated and caused reinfection in an already carious tooth, leading the tooth to be nonvital, which in turn caused the formation of a cyst. Over-retention of teeth was associated with a cystic formation of a peri-radicular cyst, which was caused due to the earlier trauma to carious primary teeth at the same site.

A peri-radicular cyst, also known as a radicular cyst, is one kind of odontogenic cyst that develops at the root of a tooth in response to an inflammatory process. An odontogenic cyst with a radicular and periapical origin is more common in the permanent dental arch and less common in primary dentition. Radicular cysts can arise as a result of pulp treatment failure in the primary teeth or as an apical infection brought on by caries.

The epithelial remnants of the periodontal ligament give rise to radicular cysts due to inflammation and the subsequent infiltration of inflammatory cells, which is typically a result of pulp necrosis. The apex of the afflicted tooth is typically involved in these cysts [6].

Dental trauma to the jaws and/or primary teeth can impede the formation of permanent teeth in 12-69% of cases [7]. The impact force acting on the bone tissue, the permanent tooth germ, or even the mechanical impact of the original tooth apex on its permanent replacement could have caused these modifications at the scene. It is also important to consider the changes brought about by medium- and long-term post-traumatic sequelae [8].

 Many causes of persistence include successor teeth transmigration or translation, impaction of permanent teeth, and congenitally absent successor teeth in those with persistent primary teeth. Pathologies such as tumors, cysts, and odontomas beneath the primary teeth can impair the development of subsequent teeth. In addition, partial or complete microdontia of the permanent teeth and trauma can also have detrimental effects [3]. There are various ways to treat retained primary teeth, like prosthesis restoration, periodontal therapy, extraction, filling, and root treatment.

Anomalies in the primary teeth can result in malocclusion, greater vulnerability to caries, and an ugly appearance of the affected teeth. Irregularities in the primary dentition show irregularities in the permanent teeth [9,10]. Prompt action would reduce issues with permanent teeth.

The treatment approaches in such conditions include the following: For patients aged between zero and three years, observation, extraction, and marsupialization, in some cases, are performed. In patients in the age group of three to six years, the treatment is extraction and marsupialization if the cyst is large and follow-up. Children six to 12 years of age should be treated with extraction, enucleation if required, and space maintainers. Endodontic treatment and orthodontic evaluation should be done in adolescents with 12+ years of enucleation and curettage. General considerations include antibiotics, analgesics, and regular follow-up.

In this report, we are studying the etiology, complications, and treatment of retained primary teeth in a male patient, seven years old, who came with a chief complaint of swelling and pain in the anterior region of the maxilla.

## Case presentation

**Figure 1:**
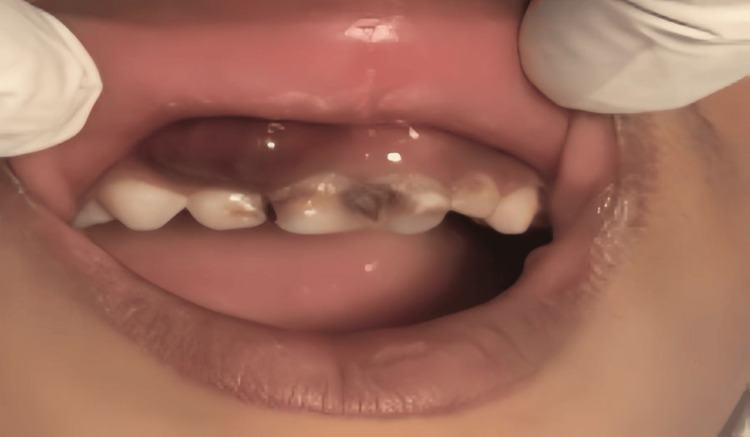
Preoperative image: Clinical presentation of the seven-year-old child with swelling at the labial aspect in the upper right anterior region of the maxilla.

A seven-year-old male patient came with a chief complaint of pain for one month, the pain was sudden in onset, sharp shooting type, and intermittent; it used to aggravate on mastication and relieved on its own. The patient also complained of pain for the past one to two days, which was dull aching and continuous and was irreversible in type and relieved on medications. It was associated with swelling, which was spontaneous and present over the upper right anterior region for two to three days. The swelling was insidious in onset and gradually progressed to the present size. On extraoral examination, the lip was competent, but there was asymmetry. On the intraoral examination, labial swelling was dark reddish brown in color, oval in shape, soft, fluctuant, non-pedunculated, diffuse, firm, non-tender, purulent, immobile extending from alveolar mucosa to border of marginal gingivae at the labial aspect of the 51, 52, 53 region with a dimension of 3 × 2 cm. No expansion of the palatal cortical plate was seen. Intraoral periapical radiograph revealed a well-defined periapical radiolucency at the 51, 52, 53 region with teeth buds of 11, 12. The patient had a history of trauma in the upper anterior region due to a fall while playing when he was five years old, and medications like antibiotics and analgesics were given at that time to manage pain and inflammation. After two years, the patient is presented with an upper scenario. Intraoral examination showed swelling at the labial aspect in the upper right anterior region of the maxilla with carious 51, 52 (Figure 1).

**Figure 2:**
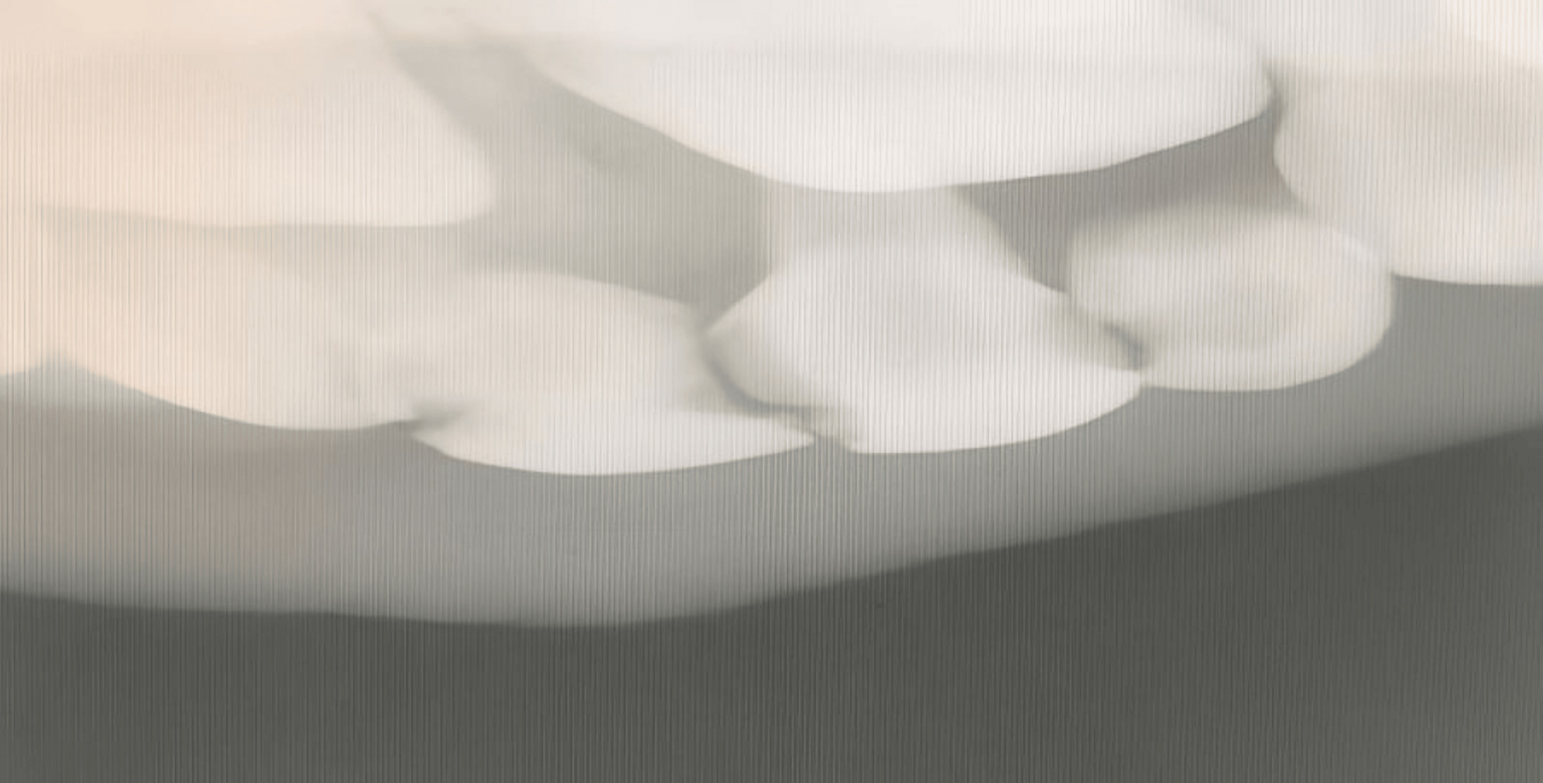
Intraoral periapical radiograph (IOPA) showing the right maxillary anterior region of the jaw. IOPA: intraoral periapical radiograph

The intraoral periapical radiograph displays the maxillary anterior region of the jaw. Teeth 51, 52, 53, and 61 are fully visible, while tooth 62 and the teeth buds of 11 and 12 are partially visible. Periapical radiolucency was observed in teeth 51, 52, and 53 (Figure 2).

Treatment plan

Following the identification of primary tooth roots and evaluation of their health, a plan of treatment was developed that included extracting the retained deciduous teeth. Consent of the parents or guardian of the patient was taken. The medical history of the patient was negative. Extraction of teeth 51 and 52 was decided from the inference of intraoral periapical radiograph. Extraction was done using infiltration by local anesthesia (2% lignocaine with adrenaline). The pus was drained, no sutures were given, and labial cortical plate expansion was taken care of by compressing via digital pressure. Irrigation of the surgical site was done using betadine (povidone-iodine) solution, medications like antibiotics and painkillers were given, and betadine mouthwash was given for seven days. Post-extraction instructions were given. The patient was recalled after three days. Eruption of teeth 11 and 12 was observed following a year of follow-up.

**Figure 3:**
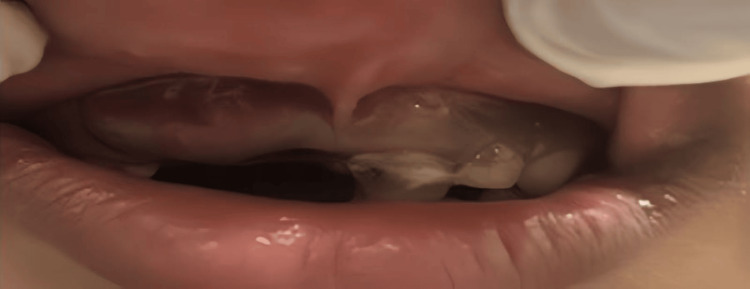
Post-operative image: Clinical picture of the site after the extraction of the retained primary teeth.

**Figure 4:**
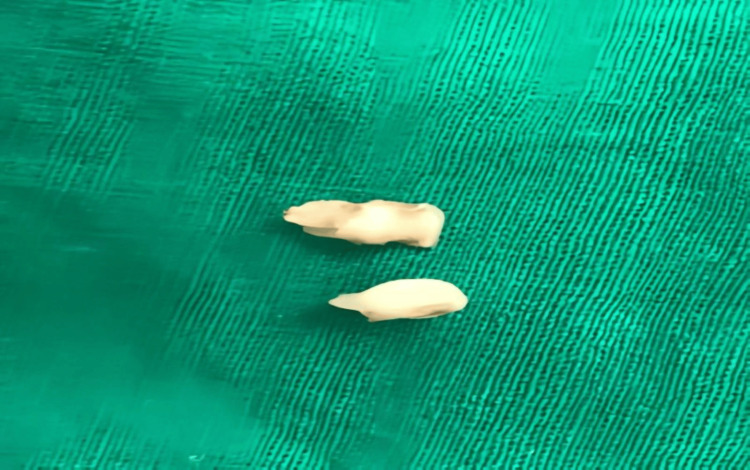
Showing extracted teeth 51 and 52.

Based on the patient's history and clinical examination, a provisional diagnosis of retained primary teeth with peri-radicular lesions was made. The treatment plan was extraction. Figure 3 shows the post-operative image, i.e., the image after the extraction. Figure 4 shows the extracted primary teeth.

## Discussion

Trauma to the primary teeth is common, out of which anterior teeth are more prone to it. Thirty percent of the children in a retrospective Danish research had experienced dental trauma before the age of seven [11], However, despite this, research in the field of dental traumatology has paid little attention to trauma to the primary dentition.

The most common primary tooth to sustain damage is the right central maxillary incisor, and pulp necrosis is the most prevalent result of invasive injury. A permanent tooth develops abnormally when a primary tooth sustains severe damage [12]. The standard of care in many of these situations is tooth extraction [13]. Table 1 presents various dental traumas in primary teeth with their treatment.

**Table 1:** Various dental traumas in pediatric patients with treatment. Reference: [14]

Trauma	Type	Treatment
Crown fracture	Enamel fracture	Smooth and polish the rough edge.
	Enamel dentin fracture	Restore with resin composite or glass ionomer cement.
	Complicated crown fracture	Pulp therapy if the pulp is exposed.
Root fracture		Stabilization, monitoring, or in some cases extraction.
Luxation displacement injuries	Subluxation	Monitor and provide a soft diet.
	Lateral luxation	Reposition the tooth gently and stabilize it.
	Extrusive or intrusive luxation	Reposition under local anesthesia; monitor for the pulp if necessary.
	Avulsion	Replant immediately if possible; otherwise store in an appropriate medium, e.g., milk, saliva, and seek immediate dental care.

Two kinds of retained deciduous teeth are seen in clinical practice: those with successors and those without. When there is no successor, radiographs of deciduous teeth show unevenly contoured roots. Lamina dura of the bone is difficult to trace. It looks like those roots are resorbed. These teeth are very difficult to extract, frequently breaking into many pieces [15].

Etiology for the condition can be classified into three categories: genetic, environmental, and systemic. When it comes to environmental factors, pathology, space constraints, crowding of the arch, and tooth bud rotation all play a part in the delayed exfoliation of primary teeth. When radiography indicates that there are typically more teeth than present, unerupted teeth may be the result of insufficient eruptive force [16].

When the permanent tooth is present but does not erupt, the primary tooth may sometimes stay in its natural position. This may be brought on by trauma that results in ankylosis of the primary teeth, the existence of extra teeth, or other impediments. Many of these problems can be fixed with orthodontic or surgical intervention [2].

In various studies done recently, it was found that post-traumatic problems can harm the tooth and its supporting components, and they can develop gradually over time. In addition to soft-tissue injuries or bone fractures, those could include root resorption, pulp canal obliteration, or pulp necrosis, with pulp necrosis being the most frequent post-traumatic consequence [17]. The findings of the study by Sabine Sennhenn-Kirchner indicate that enamel hypoplasia was brought on by an injury sustained during the tooth's maturation phase. The dental malformations, which accounted for up to 50% of the patients he examined and included ankylosis-related termination of root development or retention, were clinically more significant. Long-term clinical observation verified this. Only radiography can identify the various impacts on permanent teeth after a few months pass; clinical evaluation may not even be possible until the clinical crown has erupted [18]. The most prevalent radiographic sequela was found to be periapical radiolucency, which was linked to the finding that pulp necrosis is the most common post-traumatic outcome of deciduous teeth [19].

A carious, non-vital, discolored, or cracked tooth is typically linked to a peri-radicular cyst, also called a radicular cyst. Of all the cysts that infect the human jaw, radicular cysts make up between 52% and 68%. There is a reported age range of three to 19 years for radicular cysts originating from deciduous teeth, with a predominance in males. Mandibular molars (67%) and maxillary molars (17%) are the deciduous teeth that are most frequently affected, followed by anterior teeth. In our case, the radicular cyst was associated with traumatized anterior teeth [20].

General treatment for the peri radicular cyst aims to eliminate the source of inflammation and infection and involves the following: 1) Root canal treatment: This is typically the first line of treatment to remove the necrotic pulp and disinfect the root canal system. 2) Marsupialization and cyst enucleation: In cases where the cyst is large or persists after root canal treatment, surgical removal (enucleation) may be necessary. 3) Extraction if required practically. 4) Follow-up: Regular monitoring to ensure healing and resolution of the cyst.

In patients with retained primary teeth due to trauma to the carious anterior teeth, which eventually developed peri-radicular cyst thus causing difficulty in the development of permanent tooth buds, recent advances and treatment modalities are minimally invasive techniques, improved diagnostic tools, and interdisciplinary approaches. Diagnostic assessment includes clinical examination, radiographic imaging like cone beam computed tomography, and digital radiography. Treatment modalities include infection control using antibiotics and analgesics, surgical intervention like extraction of affected primary teeth, enucleation, curettage, regenerative and restorative approaches like bone grafting, use of space maintainer if the primary teeth are extracted to preserve the space for proper eruption and alignment of permanent teeth and orthodontic evaluation, regular check-up, and radiographic monitoring to assess the development of permanent teeth.

Preventive measures include good oral hygiene practices like regular brushing and flossing; a dietary recommendation that is a diet rich in fruit, vegetables, and dairy products to promote strong teeth and gum; limiting sugary snacks and beverages to reduce the risk of caries; and regular dental visits.

By integrating these advances and preventive measures, dental professionals can effectively manage such cases.

There are two scenarios for the case: first, the primary teeth are present with a successor, and second, the primary teeth are present without a successor. In this case, primary teeth are present with successor tooth buds, thus showing that there is no delayed eruption of permanent teeth.

In the present case scenario, there is over-retention of primary anterior teeth, due to trauma that was left untreated two years back; the teeth affected were already carious and trauma added up with reinfection, thus causing pulpal necrosis and nonvital tooth, which in turn led to inflammatory odontogenic cysts, i.e., peri-radicular cyst in the region, thus causing swelling and pain. On the radiographic assessment, the presence of a permanent tooth bud was seen. This shows that primary teeth were retained with successors and trauma-causing cystic formation (that is pathology under the primary tooth) that results in the impaction of successor teeth. Treatment for the same prevented further developmental disturbance in developing permanent tooth buds.

Such conditions can often lead to Turner’s hypoplasia, a periapical inflammatory illness in the primary tooth that causes enamel defects in the underlying permanent teeth, known as Turner’s tooth [21]. Traumatic hematoma can also be a differential diagnosis for the condition. This case is a rare occurrence in children and has multiple clinical implications. It demonstrates that even minor trauma can lead to significant conditions, emphasizing the importance of not neglecting any trauma. In treating such cases, attention should be given to both hard and soft tissues. This study presents a conservative and straightforward approach to managing children with retained deciduous teeth and peri-radicular lesions.

In the case discussed above, extraction was the simplest way to subside the pain and swelling that was due to retained deciduous teeth. The treatment for this case could have involved complex surgical intervention. Instead, we opted to extract the tooth and wait for the permanent tooth to erupt, thereby simplifying the treatment. This timely intervention prevented potential orthodontic complications that could have arisen in the future. There are various factors as well causing the retention of primary teeth. In the above case, persistence was caused due to untreated trauma and negligible behavior of guardians or parents toward the child's oral health.

## Conclusions

Nowadays, various cases come across with retained primary teeth. There are various causes of persistence involving environmental and genetic factors or disturbances in the development process. The etiology of various such conditions is not known yet even in the literature. In this case scenario, the retention of primary teeth occurred due to a trauma that aggravated the infection of carious anterior teeth, leading to pulp necrosis and causing peri-radicular cyst with pain and swelling. Peri-radicular cysts are usually associated with either treated or decayed primary teeth, or the patient refers to a history of trauma. In the case discussed above, there are retained primary teeth with peri-radicular lesions due to trauma. Various treatment modalities like extraction of tooth, marsupialization, and enucleation can be used. However, the primary treatment for the situation is the extraction of the tooth. Marsupialization may be used, but a cyst was not that major to do marsupialization. AEnucleation represents a more radical approach, which often can cause damage or lead to the removal of an unerupted successor. Due to such conditions, the extraction of the retained teeth is a suitable treatment option. Prompt diagnosis is crucial, as extracting the affected primary tooth requires managing the residual space to ensure the proper alignment of the corresponding permanent teeth. That is why routine dental check-ups and radiographic monitoring are essential to look for the development of permanent teeth. In the case discussed in this report, the simple management was the extraction of retained deciduous teeth was done using local anesthesia, thus causing less harm and pain to the young patient.
